# Association between redox biomarkers, DNA damage and aerobic capacity before and after physical stress in young men

**DOI:** 10.1016/j.redox.2025.103764

**Published:** 2025-07-10

**Authors:** Daniela Weber, Jonas Thimm, Tamina Seiz, Bastian Kochlik, Jana Raupbach, Alexander Bürkle, Tilman Grune, Markus Gruber, Maria Moreno-Villanueva

**Affiliations:** a Department of Molecular Toxicology, German Institute of Human Nutrition (DIfE), Potsdam-Rehbrücke, 14558, Germany; b Human Performance Research Centre, Department of Sport Science, University of Konstanz, 78457, Konstanz, Germany; c Human Study Center for Consumer Health Protection, Department Food Safety, German Federal Institute for Risk Assessment (BfR), 10589, Berlin, Germany; d Technical University Braunschweig, Institute of Food Chemistry, 38106, Braunschweig, Germany; e Molecular Toxicology Group, University of Konstanz, 78457, Konstanz, Germany; f German Center for Cardiovascular Research (DZHK), Partner Site Berlin, Potsdamer Straße 58, Berlin, Germany; g Institute of Nutritional Science, University of Potsdam, Potsdam, 14469, Germany

**Keywords:** Redox biomarkers, Reactive dicarbonyls, DNA damage, Aerobic capacity

## Abstract

The interaction of reactive molecules with proteins, lipids and carbohydrates results in the formation of compounds generally called redox biomarkers. It is widely recognized that high intensity exercise results in an increase of oxidative stress which in turn induces DNA damage. However, aerobic trained individuals seem to be less affected than untrained individuals. We previously showed that exercise-induced DNA damage is indeed higher in untrained individuals compared with trained individuals. But to which extent redox biomarkers are associated with DNA damage and how both are associated with aerobic capacity remains unclear. Therefore, we measured well-established redox biomarkers in plasma from young healthy volunteers before and after exhaustive exercise. We found that aerobic capacity, as measured by the level of VO_2_ peak, is negatively associated with glyoxal, methylglyoxal and 3-deoxyglucosone concentration in plasma before and after exhaustive physical exercise. In contrast, protein carbonyls, 3-nitrotyrosine and malondialdehyde were not associated with aerobic capacity. Interestingly, glyoxal was positively associated with DNA strand breaks in immune cells before but not after exhaustive exercise, indicating a beneficial effect of a high aerobic capacity on DNA integrity. These results provide a potential mechanism of how exercise protects against cardiovascular diseases, diabetes and cancer development.

## 1 Introduction

There is solid scientific evidence that demonstrates the role of physical activity in the prevention and treatment of several diseases [[1], [2], [3]]. For instance, regular physical activity improves blood glucose control and combined with modest weight loss lowers type 2 diabetes risk by up to 58 % in high-risk populations [4]. Thus, moderate to vigorous exercise is an independent treatment that can prevent, delay or reverse type 2 diabetes and is therefore recommended as a key therapy [5]. Furthermore, regular exercise that meets or exceeds the current physical activity guidelines [6,7] is associated with reduced risk of coronary artery disease, stroke, heart failure, cardiovascular disease-related mortality, and all-cause mortality [8]. Paradoxically, during exercise, skeletal muscles generate reactive oxygen (ROS) and reactive nitrogen species (RNS) that can result in oxidative damage to cellular macromolecules [9]. This incongruity might be explained by the hormesis theory, which states that a single bout of intensive exercise increases while regular exercise decreases the oxidative challenge to the body [[10], [11], [12], [13], [14], [15]]. For instance, moderate level of ROS production during exercise promotes positive physiological adaptation in the active skeletal muscles (e.g., mitochondrial biogenesis, synthesis of antioxidant enzymes, and stress proteins), while high levels of ROS production results in damage to macromolecular structures (e.g., proteins, lipids, and DNA) [16]. Furthermore, ROS are not only produced by muscles but also by other tissues, including blood cells. Recently, the contributions of neutrophils and macrophages to ROS after exhaustive exercise have been reported [17]. It is then not surprising that redox biomarkers have been proposed to assess and monitor the effects of acute training periods on physical performance [[18], [19], [20], [21]].

Redox biomarkers are molecules that respond to oxidative stress [9,22]. These biomarkers can be quantified by means of the production of ROS and RNS, levels of antioxidants, oxidation products, and the antioxidant/pro-oxidant balance [9]. Protein carbonyls (PC) and 3-nitrotyrosine (3-NT) are biomarkers of protein oxidation [23], malondialdehyde (MDA) is a biomarker of lipid peroxidation [24] and reactive dicarbonyls including methylglyoxal (MGO), glyoxal (GO) and 3-deoxyglucosone (3-DG) are formed through the Maillard reaction, the polyol pathway, glycolysis, lipid peroxidation or glucose autoxidation [25]. PC, 3-NT, MDA and reactive dicarbonyls have been extensively investigated in the context of exercise [24,[26], [27], [28], [29], [30], [31], [32], [33], [34], [35], [36], [37], [38]]. For instance, PC concentration is elevated by cycling exercise performed at 70 % VO_2_ peak, is greater following longer duration rides, and decreases within 1 h following exercise [26]. Also a single bout of strenuous squatting and sprinting results in elevated PC [27]. Gorini et al. suggest that regular physical activity protects against protein carbonylation due to the activation of the antioxidant defense or the turnover of PC [29]. In another study soccer players performed 10 consecutive training programs. MDA level did not change at the beginning but it was increased at the end of training program [33]. Furthermore, three months of moderate-intensity exercise reduced plasma 3-NT in rheumatoid arthritis patients [35] and lifelong exercise training was associated with lower dicarbonyl stress as measured by MGO and 3-DG [37].

Impaired cellular redox mechanisms are a major source of DNA damage because redox homeostasis regulates key proteins involved in DNA repair [39]. Therefore, redox biomarkers are often associated with DNA damage. For instance, MDA reacts with DNA bases inducing DNA damage [40] and is positively correlated with DNA strand breaks in patients with metabolic syndrome [41].

In order to investigate the exercise hormesis theory, physical performance, redox biomarkers and their consequences, e.g. DNA damage and how aerobic performance may impact the production of redox biomarkers in response to a single exhaustive bout of exercise need to be measured. We have previously shown that trained individuals not only have lower amount of endogenous DNA strand breaks but also lower DNA strand breaks formation and faster DNA repair after physical exhaustion compared to untrained individuals [42]. In the present work we expanded our previous experimental approach and measured PC, 3-NT, MDA and reactive dicarbonyls before and after physical exhaustion. Thus, the major aim is to correlate the aerobic capacity of the participants to measurements of plasma redox biomarkers at rest and after a single bout of exhaustive exercise. Furthermore, we asked the question whether an increase in redox biomarkers is associated with DNA damage.

## 2 Material and methods

### 2.1 Participants and study design

Participants were recruited through flyers at the University of Konstanz. The study received ethical approval from the Ethics Committee of the University of Konstanz (Approval #25/2018, September 4, 2018 and Approval #29/2022, July 5, 2020). Exclusion criteria were known heart conditions that preclude exhaustive exercise, previous positive testing for Hepatitis B, Hepatitis C, or human immunodeficiency virus (HIV), actuated cancer treatment or long-term treatment with glucocorticoids. Due to the natural hormones fluctuation that might influences performance [43,44], women were excluded from this study. Sample size estimation was calculated using G∗Power 3.1 [45]. For Wilcoxon signed rank test (one sample) and assuming normal distribution, a type I error rate of 5 % (alpha error probability = 0.05), a level of statistical power of 95 % (1 – beta error probability = 0.95) and a medium size effect Cohen's f = 0.5, we calculate a total sample size of 47 participants. A total of 68 young healthy men were included in the study.

**Fig. 1:**
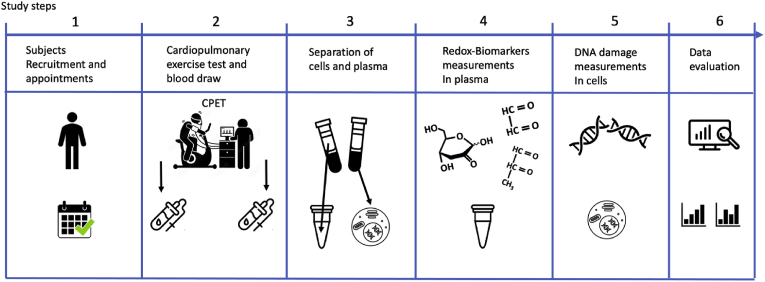
Schematic representation of the study design.

The study steps are summarized in Fig. 1. Briefly, each subject reviewed and signed consent forms before participating in the study (Fig. 1- step 1). Aerobic capacity (VO_2_ peak l/min) was evaluated during a cardiopulmonary exercise test (CPET) performing an exhaustive ramp test on a cycle ergometer. VO_2_ peak was defined as the highest value recorded during the cycling exercise since a VO_2_ plateau that defines the maximal oxygen consumption was not reached by the majority of the participants. Subjects performed the CPET test in the morning after overnight fasting and blood samples were taken before and after CPET (Fig. 1 – step 2). Blood cells and blood plasma were separated and stored until measurements (Fig. 1 – step 3). Redox biomarkers and DNA damage were measured (Fig. 1 – steps 4–5) and statistical analysis were performed (Fig. 1 – step 6).

### 2.2 Induction of physical stress (CPET test)

Prior to the exhaustive exercise anthropometric data was collected (age, body mass index (BMI), height and weight). The exhaustive exercise consisted of a cardiopulmonary exercise test (CPET) on a cycle ergometer (Ergoselect 200, Ergoline, Bitz, Germany). A ramp test was performed, and strong verbal encouragement was given until volitional exhaustion. To ensure exhaustion, heart rate and respiratory exchange rate (RER) were measured. Participants had to maintain a constant cadence between 60 and 90 rpm while load continuously increased. The ramp slope was set to 20 W/min, 25 W/min, 30 W/min, or 35 W/min, based on sex, body weight and training status. Breath-by-breath oxygen uptake and carbon dioxide emission was monitored using the Ergostik system (Geratherm Respiratory GmbH, Bad Kissingen, Germany). VO_2_ peak was defined as the highest value recorded after CPET was completed.

Directly before and 20–30 min after the ramp test around 60 mL blood was drawn. Venous blood was obtained using monovettes containing a 3.2 % citrate solution (S-Monovette® 10 mL 9NC, Sarstedt) as anticoagulant. Plasma was obtained after 1,000g centrifugation at 20 °C for 10 min. In order to extract the blood cells from the hematocrit, hematocrit was diluted with the same amount of obtained plasma. Peripheral blood mononuclear cells (PBMC) were extract from whole blood by Biocoll® (Biochrome AG, Berlin, Germany) density gradient centrifugation following the manufacturer's instructions. To determine cell concentration and viability, a CASY® Counter was used.

### 2.3 Assessment of DNA strand breaks

After PBMCs isolation, volumes of 100 μL with 4x10^6^/mL cell suspension (suspension buffer: 0.25 M mesoinositol; 10 mM sodium phosphate, pH 7.4; 1 mM magnesium chloride) were prepared. DNA strand breaks (DNA-SB) were detected by the automated FADU assay [46,47]. This assay is based on alkaline DNA unwinding in a cell lysate under controlled conditions (time, pH, and temperature) starting at DNA strand breaks. SybrGreen® (MoBiTec, Göttingen, Niedersachsen, Germany) was used as a marker to identify intact double-stranded DNA. The number of strand breaks in living cells is known to increase linearly with the radiation dose. However, the fluorescence signal intensity depends on the dose in a nonlinear fashion and displays saturation. Therefore, we previously presented a mathematical model that describes the effect of DNA unwinding on the resulting strength of the fluorescence signal and thereby captures the non-linear relationship more precisely. The fluorescence signal was then expressed as a measure of radiation dose (Gy-equivalent) using this published mathematical transformation [48].

### 2.4 Assessment of redox biomarkers

Whole blood samples in serum-separating tubes were centrifuged after collection (10 min, 4 °C, 3000×*g*) and supernatant was stored at −80 °C until analysis. The analyses of PC [49] and 3-NT in plasma was performed by in-house ELISA as described before [50]. Plasma MDA was determined by RP-HPLC coupled with fluorescence detection after derivatization with thiobarbituric acid as described by Wong et al. [51] with modifications [50]. Serum levels of dicarbonyl compounds GO, MGO, and 3-DG were analyzed as described before [52] with slight modifications [53]. Briefly, serum samples were deproteinized using perchloric acid and subsequently derivatized with *o*-phenylenediamine. GO, MGO, and 3-DG concentrations were measured using stable isotope-dilution UPLC–MS/MS (Waters, Milford Massachusetts, USA) with a run-to-run time of 8 min. Intra-run and inter-run variations were 4.3 % and 14.3 % for GO, 2.9 % and 7.3 % for MGO, and 2.4 % and 12.0 % for 3-DG, respectively.

### 2.5 Statistics

A total of 68 young healthy men were included in analysis (Table 1). Less than 2.5 % outliers were identified using the interquartile range with k = 1.5 and excluded from analyses. Data stratification and identification of outliers were performed using KNIME software [54]. Graphs, linear regression analysis and statistical tests (indicated in each figure) were performed using GraphPad Prism 10.

**Table 1:** Characteristics of the 68 participants that were included in this study. BMI = Body mass index Table 2 displays the descriptive statistics for each redox biomarker before and after exhaustive exercise.

	age (years)	height (m)	weight (kg)	BMI (kg/m^2^)	VO2 peak (l/min)
**Number of values**	68	68	68	68	68

**Minimum**	20.0	1,66	55.0	17.4	1.97
**Maximum**	35.0	1.96	143	46.6	6.31
**Range**	15.0	0.300	87.5	29.2	4.34

**Mean**	24.7	1.81	80.1	24.5	4.14
**SD**	3.32	0.0685	14.7	4.43	0.877
**SEM**	0.403	0.00831	1.79	0.538	0.106

**CV**	13.5 %	3.79 %	18.4 %	18.1 %	21.2 %

**Table 2:** Descriptive statistics of continuous variables from blood samples collected before and after exhaustive exercise (CPET). PC = protein carbonyls; 3-NT = 3-Nitrotyrosine; MDA = malondialdehyde; GO = Glyoxal; MGO = methyglyoxal; 3-DG = 3-deoxyglucosone; N = number of values.

	PC		3-NT		MDA		GO		MGO		3-DG	
	before	after	before	after	before	after	before	after	before	after	before	after
**N**	41	41	42	42	58	58	54	54	56	56	57	57
**Minimum**	0.5111	0.5662	0.0001	0.0001	0.4205	0.4163	98.58	133.9	248.1	154.1	18.25	547.6
**25 % Percentile**	0.6440	0.6311	0.5218	0.5316	0.6523	0.6915	447.8	406.6	485.6	365.2	798.0	938.2
**Median**	0.7009	0.6991	0.9264	1.066	0.7431	0.7784	622.1	670.4	702.0	655.2	1211	1415
**75 % Percentile**	0,8078	0.8526	1.454	1.431	0.9299	0.9902	1011	1393	800.1	814.1	1581	2275
**Maximum**	1.032	1.222	2.552	2.411	1.931	1.906	1336	1836	1185	1496	5085	6175
**Range**	0.5213	0.6560	2.552	2.410	1.510	1.489	1238	1702	936.7	1342	5066	5627

## 3 Results

### 3.1 Redox biomarkers before and after exhaustive exercise (CPET test)

**Fig. 2:**
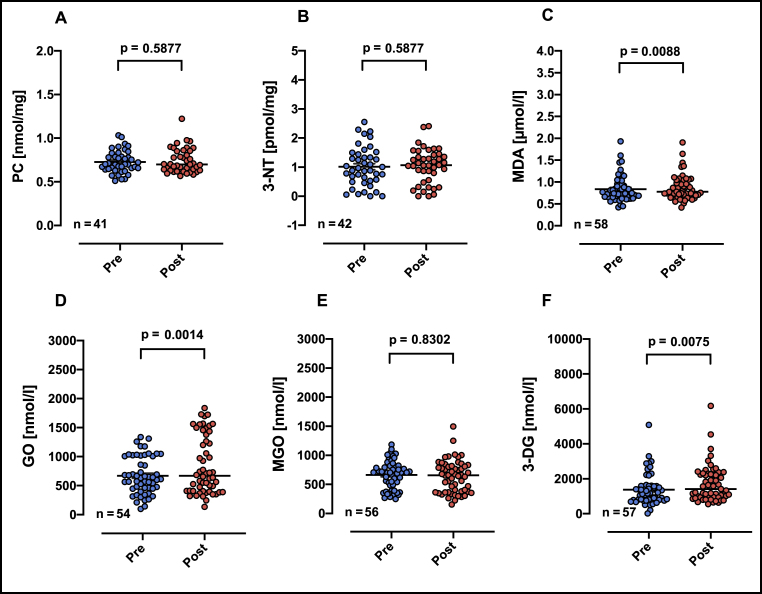
Plasma concentration of redox biomarkers in individuals before (blue dots) and after (red dots) CPET. PC = protein carbonyls; 3-NT = 3-Nitrotyrosine; MDA = malondialdehyde; GO = Glyoxal; MGO = methyglyoxal; 3-DG = 3-deoxyglucosone. Normality tests indicate not normal distribution for all biomarkers with exception of 3-NT. Therefore, p value represents statistical significance calculated from non-parametric Wilcoxon matched-pairs signed rank test, n means number of pairs included in analyses. Horizontal lines represent the median of each group.

We investigate whether high-intensity exhaustive exercise increases redox biomarkers in blood plasma. MDA, GO and 3-DG increased significantly after exhaustive exercise while PC, 3-NT and MGO did not change (Fig. 2).

### 3.2 Associations between aerobic capacity and redox biomarkers before exhaustive exercise

**Fig. 3:**
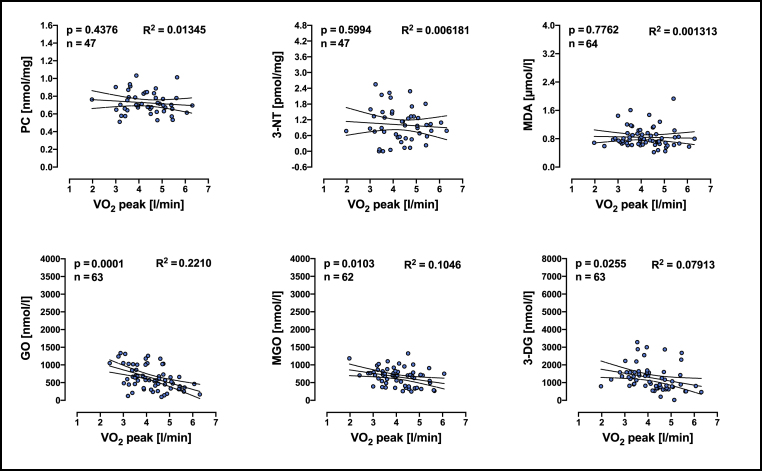
Linear regression analyses of VO_2_ peak and redox biomarkers in plasma from individuals before performing exhaustive exercise (CPET). R^2^ means goodness of fit, n means number of individuals included in the analyses. PC = protein carbonyls; 3-NT = 3-Nitrotyrosine; MDA = malondialdehyde; GO = Glyoxal; MGO = methyglyoxal; 3-DG = 3-deoxyglucosone. Linear relationship between two variables was determined by a significance test for the slope of the regression model and n means number of pairs included in analyses.

Furthermore, in order to determine whether individual' aerobic capacity have an effect on redox biomarkers, simple linear regression analyses were performed before CPET. VO_2_ peak was negatively associated with the GO, 3-DG and MGO plasma concentration before exhaustive exercise while no significant association was found for PC, 3-NT, MDA (Fig. 3).

### 3.3 Associations between aerobic capacity and redox biomarkers after exhaustive exercise

**Fig. 4:**
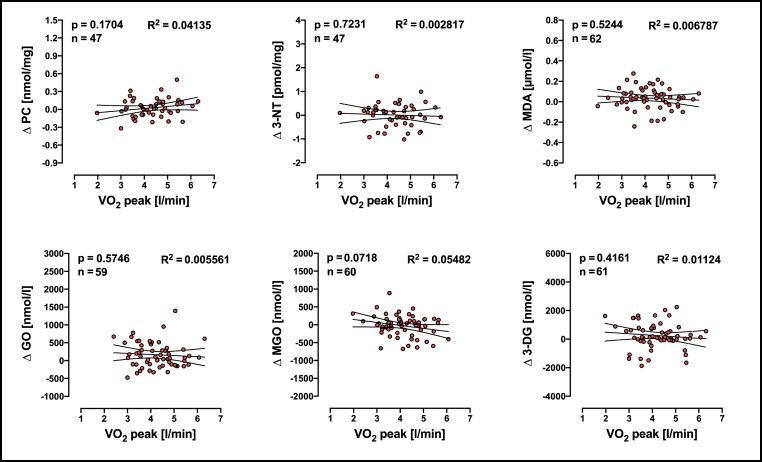
Linear regression analyses of VO_2_ peak and Δ redox biomarkers. R^2^ means goodness of fit, n means number of individuals included in the analyses. PC = protein carbonyls; 3-NT = 3-Nitrotyrosine; MDA = malondialdehyde; GO = Glyoxal; MGO = methyglyoxal; 3-DG = 3-deoxyglucosone. Linear relationship between two variables was determined by a significance test for the slope of the regression model and n means number of pairs included in analyses. Δ redox biomarkers means the difference in the redox values between before and after exhaustive exercise for each individual.

We also asked the question whether a single bout of exhaustive exercise would increase the level of redox biomarkers depending on individuals' aerobic capacity. Difference between redox biomarker values before and after exhaustive exercise (post CPET values - pre CPET values = Δ redox biomarker) was calculated before and linear regressions between VO_2_ peak and the delta (Δ) of redox biomarker were performed. There was no significant association between VO_2_ peak with any of the redox biomarkers (Fig. 4). Results presented in Fig. 3, Fig. 4 indicate that the individuals' oxygen capacity is associated to the plasma levels of dicarbonyl compounds rather than to PC, 3-NT or MDA before exhaustive exercise but not thereafter. In order to better compare our data with other relevant published studies (e.g. Refs. [10,42,[55], [56], [57]]) we have also divided the individuals in two groups defined by a VO_2_ peak median of 4.15 l/min and compared the means between the groups using mixed-effects analysis (Supplementary Fig. 1).

### 3.4 Association between DNA strand breaks and redox biomarkers

**Fig. 5:**
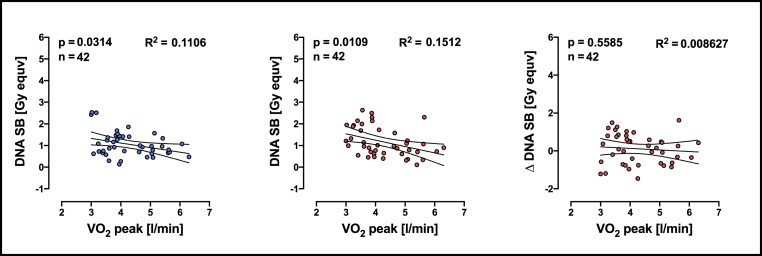
Linear regression analyses of VO_2_ peak and the number of DNA strand breaks. R^2^ means goodness of fit, n means number of individuals included in the analyses. Left panel: DNA strand breaks before performing CPET (exhaustive exercise). Middle panel: DNA strand breaks after performing CPET (exhaustive exercise). Right panel: Difference between before and after CPET in DNA strand breaks. Linear relationship between two variables was determined by a significance test for the slope of the regression model and n means number of pairs included in analyses. DNA SB means DNA strand breaks, Δ DNA SB means the difference in the DNA SB before and after exhaustive exercise for each individual (plots contained part of published data from Moreno-Villanueva et al., 2019) [42].

In our previous study, we found fewer amount of DNA-SB in individuals with higher aerobic capacity [42]. The higher the VO_2_ peak the lower the number of DNA strand breaks. This finding was significant at both time points, before and after CPET while there was no significant association between VO_2_ peak and Δ values (Fig. 5).

**Fig. 6:**
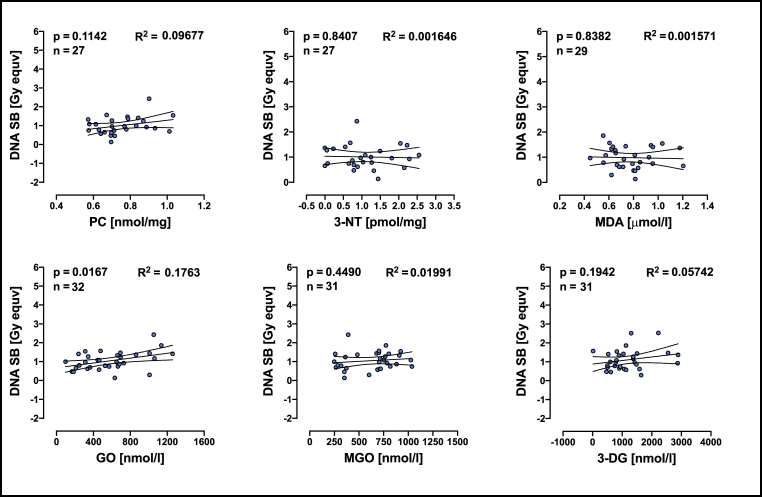
Linear regression analyses of DNA-SB and redox biomarkers measured in immune cells from individuals before performing exhaustive exercise (CPET). R^2^ means goodness of fit, n means number of individuals included in the analyses. PC = protein carbonyls; 3-NT = 3-Nitrotyrosine; MDA = malondialdehyde; GO = Glyoxal; MGO = methyglyoxal; 3-DG = 3-deoxyglucosone; DNA-SB = DNA strand breaks. Linear relationship between two variables was determined by a significance test for the slope of the regression model and n means number of pairs included in analyses.

Since VO_2_ peak appears to be associated with redox biomarkers and with DNA strand breaks we also performed regression analyses between redox biomarkers and DNA strand breaks before and after exhaustive exercise. Higher levels of glyoxal in plasma is associated with higher numbers of DNA strand breaks in cells but only before exhaustive exercise (Fig. 6). No redox biomarkers were associated with Δ DNA SB (Supplementary Fig. 2).

## 4 Discussion

While regular moderate exercise enhanced antioxidant defenses in the body [58], exhaustive exercise increased the production of reactive oxygen and nitrogen species, which in turn can cause oxidative stress [16,59]. However, there are differences between studies reporting increases, decreases, and no changes in redox biomarkers' concentration following exercise in humans, which could be explained by differences in training status and/or intensity and duration of the exercise [34,59,60]. In the present study we investigated the association between redox biomarkers, DNA damage and aerobic capacity in young men. PC, 3-NT and MDA were not associated with VO_2_ peak or with DNA-SB whereas the level of aerobic capacity was significantly correlated with plasma level of GO, MGO and 3-DG (Fig. 3, Fig. 4). Furthermore, GO was associated with the level of DNA-SB (Fig. 6). The fact that we did not find any association between VO_2_ peak and the values difference between before and after the exhaustion test indicate that the observed effects are likely a result of prolonged adaptation through training rather that an effect of a single bout of exhaustive exercise. Thus, individuals with a higher aerobic capacity have lower levels of plasma reactive dicarbonyls and low numbers of DNA-SB.

It should be noted that not only ROS itself [61] but also reactive carbonyls can directly interact with the DNA inducing DNA damage [[62], [63], [64], [65]]. Therefore, an association of all measured redox biomarkers with DNA-SB was expected. As mentioned above, PC and 3-NT and MDA are biomarkers of increased oxidative stress [23] while GO, MGO and 3-DG can be additionally formed through the Maillard reaction, the polyol pathway, glycolysis, lipid peroxidation or glucose autoxidation [25] and therefore, induction of oxidative stress is not an exclusive requirement for the formation of reactive dicarbonyls. Furthermore, the aerobic capacity VO_2_ peak of the individuals included in this cohort ranged from 1.97 to 6.31 l/min and VO_2_ peak (relative to body weight) from 25.25 to 80.9 ml/min/kg. In this cohort 58 individuals out of 68 have a relative VO_2_ peak higher than 40 ml/min/kg, which is considered above the average according with published reference VO_2_ values [66,67]. In young healthy individuals, moderate training is sufficient to keep the oxidative stress from being elevated to dangerous level upon exercise [68] which could explain the lack of association between MDA, PC, 3-NT and DNA damage in this cohort.

It also could be argued that DNA damage might be mainly associated with glucose homeostasis. Indeed, high glucose increases DNA damage and regulates the expression of multiple DNA damage response genes *in vitro* [69] and high blood glucose is associated with cellular senescence and persistent DNA damage *in vivo* [70]. Interestingly, short periods of hyperglycaemia, as occur in impaired glucose tolerance, may be sufficient to increase the concentrations of alpha-oxoaldehydes such as GO, MGO and 3-DG [71]. Mechanisms by which glucose is metabolized [72] and reactive dicarbonyls are formed [25] are known. It is also well-investigated how exercise affects glucose metabolism. A recent meta-analysis shows that exercise training reduces fasting glucose, fasting insulin, and IR index (measured by HOMA-IR) in sedentary adults without diabetes [73]. Regarding genotoxicity, high glucose increases DNA damage *in vitro* [69] which could explain the observed increase in DNA single strand breaks in lymphocytes from diabetic individuals *in vivo* [74,75]. Furthermore, the formation of endogenous aldehydes such as 3-deoxyglucosone and glyceraldehyde increase under hyperglycemic conditions and these aldehydes induce DNA damage direct, through binding to DNA, or indirect, through production of H_2_O_2_ [76], linking diabetes to cancer [77]. Interestingly, we found an association between plasma-glyoxal and DNA strand breaks in PBMCs (Fig. 6). Glyoxal is a small dialdehyde that can be transported across the cell membrane via active and passive transport [78,79] and induces damage to proteins, lipids, and nucleic acids including DNA strand breaks (DNA-SB) and protein-DNA crosslinks (DPCs) [64,[80], [81], [82]]. Especially DPCs are highly toxic since they can form steric blockades negatively impacting DNA replication and DNA transcription, as well as interfering with accessibility of DNA repair proteins [83,84]. Our previous results indicate that cells from aerobic trained individuals repair radiation-induced DNA-SB “faster” than cells from untrained individuals [42]. Here we tested whether plasma-glyoxal concentration *in vivo* is associated with DNA repair capacity in PBMCs *ex vivo*. We found that cells from subjects with higher plasma-glyoxal had a significantly lower repair capacity (Supplementary Fig. 3). Thus, maintaining low levels of plasma-glyoxal, as seeing in trained individuals, protects cells against genomic instability. Indeed, regular physical activity induces upregulation of cellular glyoxal detoxification pathways such as reduced glutathione (GSH) [[85], [86], [87], [88]] accordantly, endurance athletes have a lower levels of MGO and 3-DG [37].

**Fig. 7:**
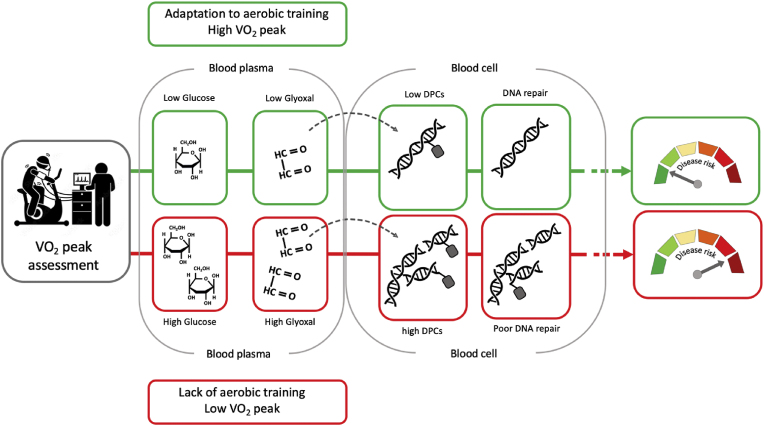
**Schematic representation of study outcomes and potential mechanisms.** Glyoxal can cross cells' membranes inducing DNA-SB and DNA-protein crosslinks. Individuals with high aerobic capacity (green frames) have a low concentration of glyoxal in blood and therefore a low level of DNA damage which can be successfully repaired. Whereas, individuals with low aerobic capacity (red frames) have a high concentration of glyoxal in blood and therefore a high level of DNA damage which can be poorly repaired increasing the risk of developing diseases.

Considering the outcome of previous studies and the data presented here, it is plausible to presume that glucose might play an important role in exercise-associated DNA integrity. More importantly, since exercise stimulates muscle glucose uptake [89] thus reducing blood glucose concentration [90], exercise programs can be applied as therapy against development of diabetes type 2, counteracting DNA damage accumulation and consequently reducing the risk of accelerated ageing and cancer development (Fig. 7).

Exercise-induced DNA damage has been often linked to oxidative stress but the association with reactive dicarbonyls have been barely investigated. Maessen and collaborators reported lower MGO and 3-DG in lifelong endurance athletes compared with sedentary controls in participants aged >45 years [37] but this study did not address DNA damage. In view of the lack of studies investigating the interplay between redox biomarkers and DNA damage in the context of exercise, our results provide novel research directions focusing on glucose homeostasis. Although our data provide some evidence, further studies, such as intervention and/or longitudinal studies, are necessary in order to strengthen the interaction between glucose and DNA damage upon exercise. Additionally, further *in vitro* experiments could elucidate the role of high insulin sensitivity in protecting DNA damage which has been far less investigated. However, this would be a different (although related) topic involving hyperglycemic (diabetic) conditions.

## 5 Conclusions

Our results indicate a strong role of aerobic capacity on lowering the plasma levels of reactive dicarbonyls. Furthermore, the association of DNA damage with GO but not with PC, 3-NT and MDA suggest that glucose metabolism, rather than oxidation processes, might be the main factor contributing to higher DNA-SB in individuals with lower aerobic capacity. Furthermore, these data emphasize the DNA damage response as a mechanism of toxicity by glyoxal which can be counteracted by aerobic physical training. But despite the statistically significant relationship between aerobic capacity, reactive dicarbonyls and DNA-SB found in this cohort, our data provide circumstantial evidence but proof that DNA-SB are induced by glyoxal is not provided. Furthermore, the inverse relationship between aerobic capacity and reactive dicarbonyls could be a consequence of one or more healthy life style factors, e.g. nutrition. Additional factors, such as the influence of exercise on the composition of immune cells in circulating blood and the antioxidative capacity need to be considered before drawing more conclusions.

## CRediT authorship contribution statement

**Daniela Weber:** Writing – original draft, Supervision, Project administration, Investigation, Data curation. **Jonas Thimm:** Writing – original draft, Validation, Methodology, Formal analysis, Data curation. **Tamina Seiz:** Methodology, Formal analysis. **Bastian Kochlik:** Writing – review & editing, Supervision, Project administration, Investigation, Data curation. **Jana Raupbach:** Writing – review & editing, Supervision, Resources, Methodology, Investigation, Formal analysis, Data curation. **Alexander Bürkle:** Writing – review & editing, Resources, Methodology. **Tilman Grune:** Writing – review & editing, Resources, Methodology. **Markus Gruber:** Writing – review & editing, Resources, Methodology. **Maria Moreno-Villanueva:** Writing – original draft, Supervision, Project administration, Investigation, Formal analysis, Data curation, Conceptualization.

## Funding

N/A.

## Declaration of competing interest

I certify that all authors have participated sufficiently in the work to take public responsibility for the content. Furthermore, each author certifies that has reviewed the manuscript and her/his stated contribution to the manuscript is accurate.

The authors declare no conflict of interest. All authors declare no financial and personal relationships with other people or organizations that could inappropriately influence (bias) their work. All authors have approved the manuscript and agree with its submission to Biomolecules.

## Data Availability

Data will be made available on request.
