# Supplementary material for: Association between redox biomarkers, DNA damage and aerobic capacity before and after physical stress in young men
Source: Redox Biol. 2025 Jul 10;85:103764. doi: 10.1016/j.redox.2025.103764 (PMC12274777; doi:10.1016/j.redox.2025.103764)
Supplement: Multimedia component 1 [file mmc1.docx]

Supplementary information

**Supplementary figure 1:** Grouped analyses of VO_2_ peak and redox biomarkers in plasma from individuals before (pre) and after (post) performing exhaustive exercise. Individuals were split in two groups by the median of VO_2_ peak: untrained (red) VO_2_ peak < 4.15 and trained (green) VO_2_ peak equals or > 4.15). Stars above the bars represent level of significance as calculated by mixed-effects analyses. PC = protein carbonyls; 3-NT = 3-Nitrotyrosine; MDA = malondialdehyde; GO = Glyoxal; MGO = methyglyoxal; 3-DG = 3-deoxyglucosone. Linear relationship between two variables was determined by a significance test for the slope of the regression model and n means number of pairs included in analyses. Number of individuals included at least 20 and not more than 30.

**Supplementary figure 2:** Linear regression analyses of Δ DNA SB and Δ redox biomarkers. R^2^ means goodness of fit, n means number of individuals included in the analyses. PC = protein carbonyls; 3-NT = 3-Nitrotyrosine; MDA = malondialdehyde; GO = Glyoxal; MGO = methyglyoxal; 3-DG = 3-deoxyglucosone; DNA SB = DNA strand breaks. Linear relationship between two variables was determined by a significance test for the slope of the regression model and n means number of pairs included in analyses. Δ redox biomarkers and Δ DNA SB means the difference in between before and after exhaustive exercise for redox biomarkers and DNA strand breaks respectively.

**Supplementary figure 3:** Linear regression analyses of dicarbonyls and repaired DNA strand breaks induced by 3.8 Gy radiation. Cells (2-4 x 10^6^ cells/ml) were irradiated *ex vivo* on ice in TexMacs Medium (Miltenyi Biotec, Germany) using a biological X-ray Irradiator X-RAD 225 iX (Precision X-Ray, Inc, North Branford, USA). The radiation time was 380 seconds at a dose rate of 0.59 Gy/min (70 kV, 30 mA, 70 cm distance, 1.25mm Al filter) resulting in a total dose of 3.8 Gy. In order to allow DNA strand breaks repair cells were incubated at 37°C for 30 minutes. DNA strand breaks were detected by the automated FADU assay. R^2^ means goodness of fit. GO = Glyoxal; MGO = methyglyoxal; 3-DG = 3-deoxyglucosone; DNA-SB = DNA strand breaks. Linear relationship between two variables was determined by a significance test for the slope of the regression model and n means number of pairs included in analyses.
